# Effect of Aerobic Exercise on Mental Health in Older Adults: A Meta-Analysis of Randomized Controlled Trials

**DOI:** 10.3389/fpsyt.2021.748257

**Published:** 2021-10-06

**Authors:** Lei Yao, Hanliu Fang, Wanchun Leng, Junfeng Li, Jindong Chang

**Affiliations:** ^1^School of Physical Education, Southwest University, Chongqing, China; ^2^Institute of Motor Quotient, Southwest University, Chongqing, China; ^3^Qingdao Mental Health Center, Qingdao University, Qingdao, China; ^4^Ministry of Sports, Shandong Technology and Business University, Yantai, China

**Keywords:** mental health, aerobic exercise, MCI, dementia, depression, cognition, older adults

## Abstract

**Introduction:** The recommendation of exercise programs in the senior population may benefit inactive and sedentary individuals and improve and help to treat specific health conditions. The purpose of this review is to summarize the published evidence from RCT studies of aerobic exercise interventions for mental health in older adults over the last 20 years.

**Methods:** A literature search was conducted using electronic databases including Web of Science, PubMed/Medline, and ProQuest.

**Results:** A total of 15 studies met the inclusion criteria. The subjects of these studies were aged 60 years or older and had various physical health statuses. In 15 studies, the mean effect size for the experimental outcome was 0.56 ± 0.39 (95%CI: 0.36–0.76). One-way ANOVA indicated no significant differences in the intervention duration [*F*_(2,15)_ = 0.919, *p* = 0.420], subject category [*F*_(2,15)_ = 0.046, *p* = 0.955], or measurement category [*F*_(3,14)_ = 0.967, *p* = 0.436]. However, there were significant differences in exercise frequencies [*F*_(2,15)_ = 6.03, *p* = 0.012].

**Conclusion:** The available evidence suggests that aerobic exercise is beneficial for improving the mental health of adults aged 60 years and older. The intervention effect can be achieved regardless of the type of subject and the duration of the intervention. Further, the present study indicates that low-frequency, long-term and regular aerobic exercise is more effective for older adults. Therefore, we recommend that older adults to exercise at a low frequency depending on their physical condition.

## Introduction

The World Health Organization (WHO) has published several cross-national comparisons of the prevalence, severity, and treatment progression of mental disorders (1–4). Studies have concluded that the 12-month prevalence of any mental disorder is highly variable. However, most countries have no access to timely treatments for mild or moderate mental disorders (5). For example, the median delay in seeking treatment for anxiety disorders is 3 years in Israel and 30 years in Mexico (5). In addition, seeking treatment for mental illness does not mean that individuals are optimally treated, and the mortality rates are higher for those with chronic or recurrent mental illnesses (6), while the morbidity is higher when depression occurs in combination with physical illnesses such as diabetes or cardiovascular disease (7). Data show that those with mental disorders die 10–15 years earlier than the general population, and major contributing factors include preventable cardiovascular diseases that are caused by poor lifestyle choices, such as a lack of physical activity (8).

Most people know that exercise and physical activity are critical for maintaining physical health; however, what about mental health? According to the U.S. Department of Health and Human Services, exercise can be defined as “physical or mental exertion, especially to train or improve health,” while, according to the U.S. Department of Health and Human Services, physical activity is “any physical exercise that exercises muscles and requires more energy than rest.” Physical activity is defined by the NIH as “any physical exercise that builds muscle and requires more energy than rest” (9). According to the U.S. DHHS, mental health can be defined as “our emotional, psychological, and social well-being. It helps determine how we handle stress, how we relate to others, and how we make choices” (Mental Health). However, it is still considered taboo to discuss mental health in the public arena. A study of 2,000 people conducted by The Guardian UK found that 30% of people found it “difficult to admit publicly that they have a mental illness” and that “admitting to a mental health condition is harder than admitting to having an alcohol problem, being broke or being gay” (9). People “are four times more likely to break up if their partner is diagnosed with major depression than if they have a physical disability” (10). Mental health has become a major “enemy” of people's health. Especially in the elderly population, the decline of various body functions due to physical decline directly affects their physical and mental health. However, physical activity is a simple and effective form of exercise, so it could play a more prominent role.

Numerous recent epidemiological studies have reviewed the relationship between physical activity and mental health (11). A meta-analysis of prospective studies including nearly 267,000 individuals showed that higher levels of PA were associated with lower odds of developing depression. In another meta-analysis including more than 80,000 people, PA was also associated with elevated odds of experiencing anxiety symptoms but lower odds of anxiety disorders (12). The data showed that, the higher the amount of PA, the lower the risk of mental health problems. There appears to be a dose–response relationship between increased PA and mental health and functioning across exercise modalities (13). Aerobic and resistance exercise proved to be of additional benefit to health (14). In conclusion, the epidemiological evidence supports the idea that more habitual PA is associated with better mental health and functioning (15).

The current research generally agrees that exercise has beneficial effects on a range of mental health outcomes. Some studies have observed that exercise improves mental health in various ways (16–18). For instance, neurobiological theories are used to explain the mechanisms by which aerobic exercise improves mental health in middle-aged and older age groups (18–20). Of these, the conceptual model of neurobiological and behavioral learning mechanisms (NBLMs) and the three overarching mechanistic hypotheses (TOMHs) are widely popular. The NBLM model assumes that exercise improves the neurobiological system of adaptive learning, as well as affective and cognitive control processes, reinforcing a virtuous circle and synergistically improving the regulation of cognitive and affective responses (20). The TOMHs comprise three hypotheses: (a) mental health is associated with the physical effects of exercise, (b) exercise improves mental health through neurobiological mechanisms, and (c) exercise is a vehicle for developing mechanisms of behavioral change (e.g., self-regulatory skills and self-efficacy). Smith et al. confirmed that the TOMHs were useful for constructing hypotheses about treatment improvements (15). However, the evidence for a dose–response effect of exercise is less robust than the observations. Although the frequency of exercise required for therapeutic mental health benefits appears to vary by population and exercise modality (21), interestingly, few studies have linked the degree of improvement to the frequency or duration of exercise (19).

The primary purpose of the current study was to review the randomized controlled trials studying the effects of aerobic exercise on older adults' mental health over the past 20 years and to analyze the effects of aerobic exercise (and their differences) on the effectiveness of mental health interventions in older adults, to provide scientific assurance that older adults should participate in aerobic exercise.

## Methods

### Search Strategy

The literature for this study was identified by conducting a comprehensive search in electronic databases, including Web of Science, PubMed/Medline, and ProQuest. The search period ranged from January 2000 to December 2020. The keywords used in our searches were exercise, aerobic exercise, mental health, mental illness, and mental disorders. After removing duplicates, the titles and abstracts of the retrieved references were screened to exclude articles that did not meet the inclusion criteria (22). The full texts of the remaining articles were obtained and fully assessed by the authors (LY and JL). The reference lists of the final included articles were also screened to identify additional studies. The decision to include disputed articles was made jointly with the corresponding author (JC).

### Selection Criteria

Studies were considered for inclusion if they met the following criteria (23): (1) the article was written in English; (2) a randomized controlled trial design was used to compare the aerobic exercise intervention group with a control group (either daily life or other forms of exercise); (3) the research question involved cognitive or mental health; (4) the study subjects were 60 years of age or older; and (5) the effect of aerobic exercise on the subjects' mental health was assessed. Studies were excluded if (1) the study subject was completely unable to care for himself/herself (had a severe physical disability); (2) the study design included other types of interventions (e.g., intervention diets); or (3) the study results did not include a cognitive or mental health component.

### Risk-of-BIAS Assessment

A risk-of-bias assessment was performed to ensure the rigor of the sources of evidence. According to the PRISMA-Scr guidelines, we conducted a partial risk-of-bias assessment based on the Cochrane Guidelines (21). The Cochrane Risk of Bias Tool was used on Review Manager 5.4 (https://community.cochrane.org). Two reviewers independently assessed the sequence generation, allocation concealment, blinding of participants, blinding of assessors, incomplete outcome data, and selective outcome reporting for the included studies (21).

### Data Extraction and Analysis

Data were extracted from each article using a pre-designed template according to the study design, sample characteristics, measures, intervention duration, intervention design, and intervention effects (22). The randomized controlled trials (RCTs) had to distinguish between two and three groups in their designs. The specific headings of the summary table included the author (as well as the year of publication and country where the study was conducted), subjects' health characteristics, sample size, mean or age range of the sample, measure/intervention involving aerobic exercise, and intervention effect size (ES). If the study provided values for the intervention effect sizes, the data were extracted directly. If the study did not directly provide values for the effect size, conversion was performed using means, standard deviations (standard errors) and sample sizes; F-values and sample sizes; or *t*-values, *p*-values and sample sizes. Specific conversions were performed using an online program developed by Wilson (24). Additionally, Cohen's d shows a large bias when the sample is small (<20 for the overall sample or <10 for each group). Therefore, Cohen's d calculated based on small samples needs to be corrected using a method proposed by Hedges and Olkin (25). Descriptive statistics and one-way ANOVA were performed on the extracted data using the SPSS 24.0 software.

## Results

### Selection of Sources of Evidence

**Figure 1:**
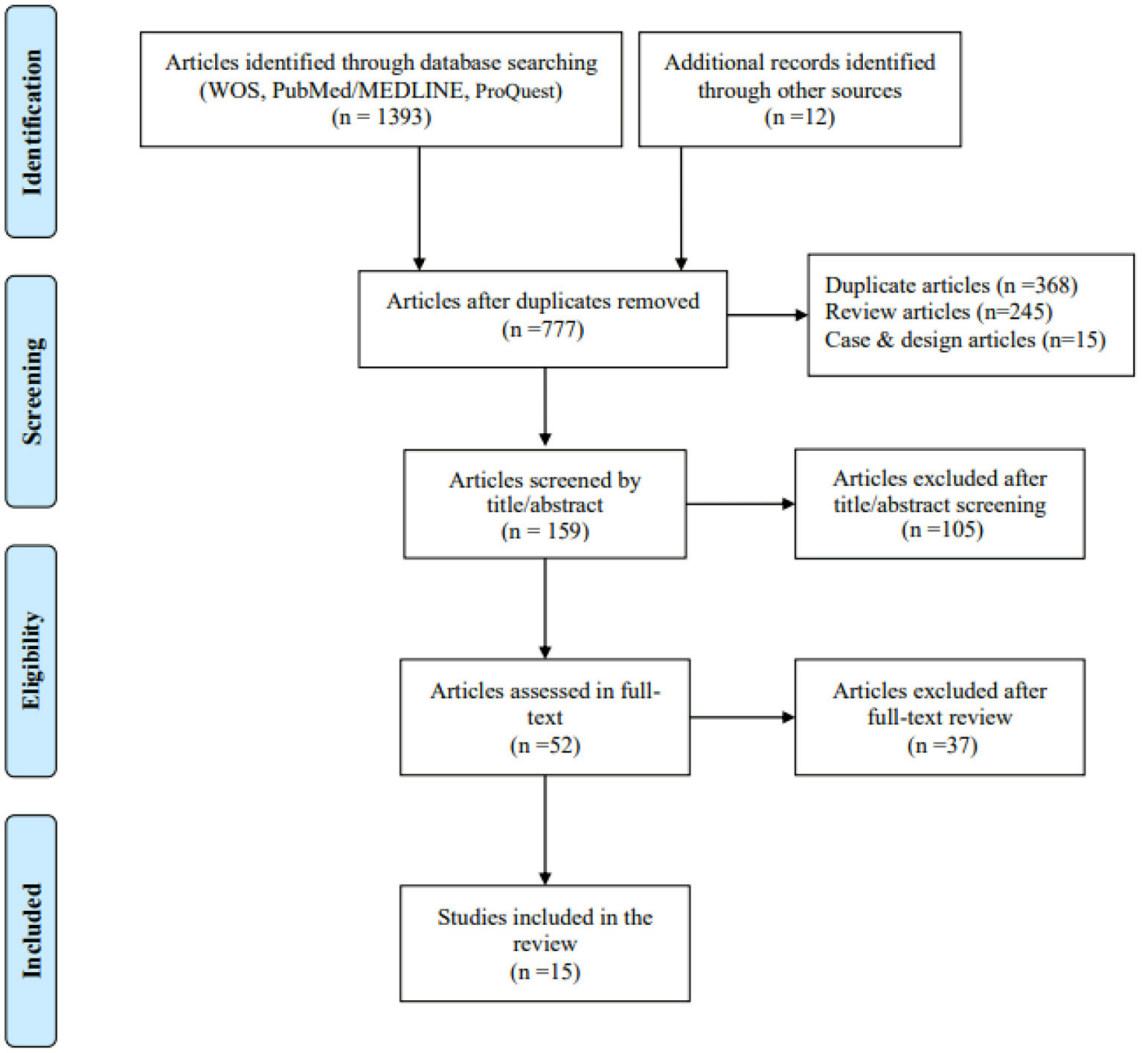
Flow diagram for evidence source search. From: Moher et al. (26).

A total of 1,393 articles were identified using electronic databases such as Web of Science, PubMed/Medline and ProQuest, as were 12 articles from other systematic reviews. After removing duplicates and reviewing the titles, abstracts and full texts, 15 studies were finally included in the present study (Figure 1). Of these studies, two reported two and three measures of testing, respectively. Thus, a total of 18 intervention-effect-size results needed to be extracted.

### Characteristics of Sources of Evidence

Data from 1,487 participants from 15 studies were included in the evidence analysis (see Table 1). Overall, the mean age of the participants was a minimum of 66.43 years and a maximum of 83.59 ± 7.05 years, with five studies (29, 31, 35, 37, 40) in which the subjects were over 65 years of age, the rest being over 60 years of age. The duration of the exercise interventions was at least 8 weeks (2 months) and at most 15 months. The frequency of the exercise interventions was 2–7 times per week, with the frequency of those in the majority of the studies being 3–5 times per week. In addition, five studies specified maximum loads for exercise, with the load controlled at 50–75% of the maximum heart rate (27, 33, 34, 39, 40); three studies also emphasized that subjects' attendance had to be no <70 or 80% (29, 37, 39).

**Table 1:** The characteristics of sources of evidence.

**ID**	**Name, country**	**Time**	***N* (f)**	**Subjects**	**Age**	**RCT**	**Measure**	**Exercise design**	**Exercise frequency**
1	Anderson-Hanley et al. (11) USA	3 m	111 (73)	MCI	All: 78.1 ± 9.9 EG1: 80.9 ± 12.3 EG2: 75.4 ± 9.83	RCT three groups	Stroop A/C	EG1: exer-tour, low cognitive load, virtual scenic bike tour EG2: exer-score, high cognitive demand, videogame CG: game-only	EG1: 20–45 min, 3–5 tim/wk EG2: 20–45 min, 3–5 tim/wk CG: N/R
2	Awick et al. (27) USA	12 m	179 (N/R)	Low-active non-CI	All: 66.4	RCT	SF12-MCS	EG: walking CG: strengthening and flexibility	EG: first 7 weeks, 50–60% HR; next, 65–75% HR CG: 4 muscle resistance, 2 balance, 1 yoga, and 1 exercise of self-choice in each class
3	Bieler et al. (28) Denmark	4 m	152 (103)	OA	EG1: 69.6 ± 5.4 EG2: 70.0 ± 6.3 CG: 69.3 ± 6.4	RCT three groups observer-blinded	SF36-MCS	EG1: NW, Nordic Walking EG2: ST, strength training CG: HBE, home-based exercise	EG1: NW, 1 h × 3 tim/wk, 12–14 on the Borg scale EG2: ST, 1 h × 3 tim/wk, 75% of 1RM CG: HBE, exercises recommended by the DAA
4	Cancela et al. (29) Spain	15 m	189 (126)	Dementia	EG: 80.63 ± 8.32 CG: 82.90 ± 7.42	RCT blinded	CSDD	EG: aerobic exercise CG: not exercise	EG: daily cycling sessions; 15 min/s, >70% monthly attendance; CG: daily life
							MEC		
							NPI		
5	Cheung et al. (30) USA	8 w	73 (73)	OA	EG1: 68.9 ± 7.1 EG2: 74.4 ± 7.5 CG: 71.8 ± 8.0	RCT three arms blinded	SF12-MCS	EG1: HY, yoga EG2: ASE, aerobic and strengthening CG: education	EG1: HY, (a) 45 min/wk × 8 wk, and (b) 30 min/day, 4 tim/wk of yoga EG2: ASE, (a) 8 tim/wk, (b) aerobic 15–30 min/day, 4 tim/week, and (c) strengthening 30 min/day, 2 tim/wk CG: education brochures, weekly telephone
6	Eggenberger et al. (31) Switzerland	6 m	89 (46)	Non-CI	EG1: 77.3 ± 6.3 EG2: 78.5 ± 5.1 CG: 80.8 ± 4.7	RCT three groups blinded	PACES	EG1: DANCE, virtual reality video game dancing EG2: MEMROY, treadmill walking with simultaneous verbal memory training CG: PHYS, treadmill walking	Each group training: 2 tim/wk, 1 h/tim, vigorous intensity
7	Hall et al. (32) USA	12 w	54 (5)	PTSD	EG: 67.7 ± 3.2 CG: 66.9 ± 4.3	RCT-Pilot blinded	SF36-PCS	EG: exercise CG: wait-list usual care	3 tim/wk, >150 min/wk, moderate intensity
8	Karssemeijer et al. (33) Netherlands	12 w	115 (54)	Dementia	EG1: 80.9 ± 6.1 EG2: 79.0 ± 6.9 CG: 79.8 ± 6.5	RCT: three arms	EFIP	EG1: stationary cycling training EG2: cognitive-aerobic bicycle training CG: relaxation and flexibility	EG1: 30-50 min/tim, 3 tim/wk, 65–75% HR EG2: 30–50 min/tim, 3 tim/wk, 65–75% HR CG: 30 min/tim, 3 tim/wk
9	Langoni et al. (34) Brazil	24 w	52 (40)	MCI	EG: 72.6 ± 7.8 CG: 71.9 ± 7.9	RCT, single-blinded	MMSE	EG: group exercise (aerobic and strength)CG: no exercise	EG: 60 min/tim, 2 tim/wk, 60–75% HR CG: N/R
10	Middleton et al. (35) Canada	12 w	126 (82)	cognitive complaints	All: 73.0 ± 6.0 EG: 72.5 ± 5.9 CG: 74.3 ± 5.9	RCT: blinded 2 × 2 factorial design	SF12-MCS	EG: aerobic or stretching/toning,CG: mental activity, computer-based cognitive training or educational DVDs	EG: 3 × 60 min/wk CG: 3 ×60 min/wk
11	Parvin et al. (36) Iran	12 w	32 (N/R)	AD	All: 67.4 ± 8.8	RCT, single-blinded	MoCA	EG: visual stimulation (muscle endurance, balance, flexibility, and aerobic exercises)CG: daily life	EG: 2 ×40–60 min/wk, warm-up 10 min, exercises 20–40 min, cool down 10 min. CG: daily life
12	Suzuki et al. (37) Japan	12 m	50 (27)	amnestic MCI	EG: 75.3 ± 7.5 CG: 76.8 ± 6.8	RCT	MMSE	EG: aerobic exercises, muscle strength, postural balance CG: education classes	EG: 90 min/d, 2 d/wk CG: 3 d /12 mos
13	Varela et al. (38) Spain	15 m	39 (15)	Non-CI	EG: 83.59 ± 7.05 CG: 77.94 ± 8.79	RCT, single-blinded	MEC	EG: cycling CG: recreational activities	EG: self-selected intensity 15 min/d, >70% completion rate CG: 3 d /12 mos
							NPI		
14	Wanderley et al. (39) Portugal	8 m	105 (27)	Non-CI	EG1: 70.0 ± 5.7 EG2: 67.3 ± 4.9 CG: 67.8 ± 5.5	RCT three-groups blinded	SF36-MCS	EG1: aerobic training EG2: resistance training CG: daily lifestyle	EG1: AT-−70–80% HR reserve, attendance rate >80%; 3 d/wk, 50 min/d; EG2: RT-−80% 1RM, attendance rate >80%; 3 d/wk, 50 min/d; CG: WL— not to change daily lifestyle
15	Zanetidou et al. (40) Italy	24 w	121 (86)	Late-life depression	EG: 74.9 ± 6.2 CG: 75.6 ± 5.6	RCT single-blinded	Anxiety	EG1: AD + NPE (low-intensity, non-progressive exercise), mat work and instrumental exercises EG2: AD + PAE (high-intensity, progressive aerobic exercise), cycling exercise CG: AD (sertraline)	EG1: 3 tim/wk, <70% HR EG2: 3 tim/wk, <70% HR CG: daily lifestyle

*N/R, not reported; EG, experimental group; CG, control group; w, week; m, month; tim/wk, times/week; HR, heart rate. MCI, mild cognitive impairment; Non-CI, without cognitive impairment; PTSD, post-traumatic stress disorder; AD: Alzheimer's disease; OA: osteoarthritis. EFIP, Evaluative Frailty Index for Physical activity; SF12/36, SF-12/36 Health related Short Form 12/36; MCS, mental component summary; CSDD, Cornell Scale for Depression in Dementia; MEC, Mini-Examen Cognoscitive; NPI, Neuropsychiatric Inventory; PACES, Physical Activity enjoyment scale; MMSE, the mini-mental state examination; MoCA, Montreal cognitive assessment*.

All the experimental designs included in this study were performed RCTs. Of all the included studies, eight used a three-group experimental design, with two groups for the exercise intervention and one non-exercise control group. For the other seven studies, participants were randomized into two groups for the exercise intervention and control group (41). In the three-group experimental design, except for the aerobic exercise, another exercise intervention group was studied, focusing on resistance training (27), stretching training (30), or a cognitive intervention plus aerobic training (33). The subjects in the study included three categories: no cognitive impairment (27, 31, 38, 39), cognitive impairment [mild (11, 34, 35, 37), dementia (29, 33), depression (32, 40), and Alzheimer's disease (36)] and physical impairment (osteoarthritis, etc.) (28, 30).

### Risk-of-Bias Assessment for Sources of Evidence

**Figure 2:**
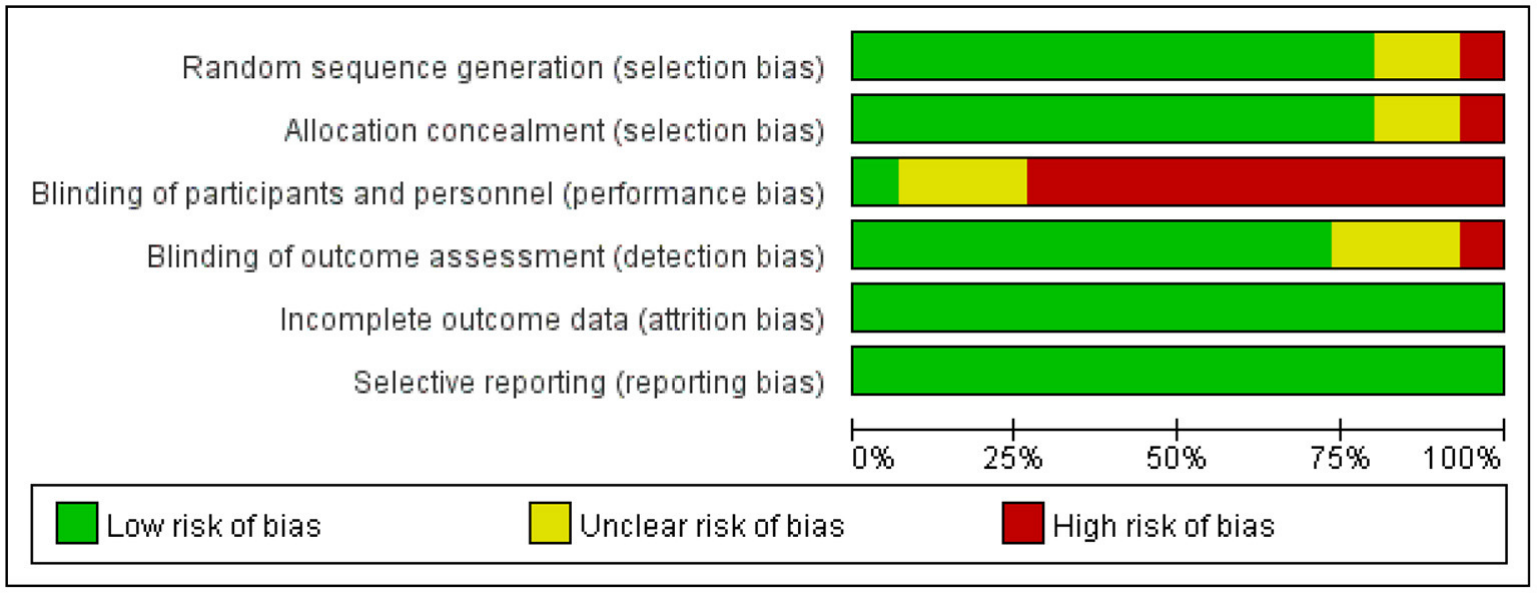
Risk-of-bias chart for studies included in the quantitative analysis.

Figure 2 shows the assessment of the risk of bias for the sequence generation, allocation concealment, participant blinding, assessor blinding, incomplete outcome data, and selective outcome reporting (21). As shown in Figure 2, 3 of the 15 studies were unclear in the sequence generation (11, 33, 37), and four, in allocation concealment (32, 37) and the blinding of the assessor (11, 27, 37). Only one study reported blinding of the participants (31). Otherwise, all the studies had a low risk of bias in all domains (for details, see the online Supplementary Table 1).

### ANOVA of the Intervention Effect Sizes

ANOVA was performed to facilitate the analysis of differences according to the various types of measures, durations, study subjects and exercise frequencies (42). We first categorized the data presented in Table 1. The measurements were coded as follows. (1) Measure category: 1 = SF12/SF36; 2 = NPI; 3 = Attitude (CSDD/EFIP/PACES/Anxiety); 4 = MMSE/MoCA/MEC. (2) Duration category: 1 = ≤3 months or 13 weeks; 2 = 3–6 months or 14–26 weeks; 3 = >6 months or 26 weeks. (3) Subject category: 1 = MCI/Dementia; 2 = Non-CI; 3 = OA. (4) Frequency category: 1 = <3 times/week; 2 = 3–5 times/week; 3 = >5 times/week.

Table 2 shows the effect sizes of the included studies and the recoded data. The mean effect size of the 15 included studies was 0.56 ± 0.39 (95%CI: 0.36–0.76). Table 3 shows the results of the descriptive statistics and one-way ANOVA for the effect sizes of the included studies. The results of the one-way ANOVA show that there were no significant differences in the intervention duration [*F*_(2,15)_ = 0.919, *p* = 0.420], subject category [*F*_(2,15)_ = 0.046, *p* = 0.955], or measure category [*F*_(3,14)_ = 0.967, *p* = 0.436]. However, there was a significant difference in the exercise frequency factor [*F*_(2,15)_ = 6.03, *p* = 0.012].

**Table 2:** Inclusion of study effect sizes and classification of indicators.

**ID**	**Author, year**	**ES (Cohen's *d*)**	**ES level**	**Measure category**	**Duration category**	**Subject category**	**Frequency category**
1	Anderson-Hanley (11)	0.52	M	4	1	1	2
2	Awick (27)	0.44	S	4	3	2	2
3	Bieler (28)	1.13	L	2	2	3	2
4	Cancela (29)	0.35	S	4	3	1	3
5	Cancela (29)	0.48	S	4	3	1	3
6	Cancela (29)	0.42	S	2	3	1	3
7	Cheung (30)	0.16	S	4	1	3	2
8	Eggenberger (31)	1.06	L	2	2	2	1
9	Hall (32)	0.48	S	1	1	1	2
10	Karssemeijer (33)	0.35	S	1	1	1	2
11	Langoni (34)	0.65	M	1	2	1	1
12	Middleton (35)	0.08	S	3	1	1	2
13	Parvin (36)	1.70	L	3	1	1	1
14	Suzuki (37)	0.74	M	1	3	1	1
15	Varela (38)	0.54	M	1	3	2	3
16	Varela (38)	0.22	S	3	3	2	3
17	Wanderley (39)	0.47	S	1	3	2	2
18	Zanetidou (40)	0.30	S	4	2	1	2

*ES Level: S, Small; M, Medium; L, Large*.

*Measure category: 1, SF12/SF36; 2, NPI; 3, Attitude (CSDD/EFIP/PACES/Anxiety); 4, MMSE/MoCA/MEC*.

*Duration category: 1, ≤3 months or 13 weeks; 2, 3–6 months or 14–26 weeks; 3, >6 months or 26 weeks*.

*Subject category: 1, MCI/Dementia; 2, Non-CI; 3, OA*.

*Frequency category: 1. <3 tim/wk; 2, 3–5 tim/wk; 3. >5 tim/wk*.

**Table 3:** Outcomes for descriptive statistics and ANOVA.

	***N***	***M***	**SD**	**95%CI**		**Min**	**Max**	***F***	***p***
				**Lower**	**Upper**				
**Subjects**									
MCI/dementia	11	0.55	0.42	0.27	0.83	0.08	1.70	0.046	0.955
Non-CI	5	0.55	0.31	0.16	0.93	0.22	1.06		
OA	2	0.65	0.69	−5.52	6.81	0.16	1.13		
**Duration (weeks/months)**									
≤13/3	6	0.55	0.59	−0.07	1.17	0.08	1.70	0.919	0.420
14–26/3–6	4	0.79	0.39	0.17	1.40	0.30	1.13		
>26/6	8	0.46	0.15	0.33	0.58	0.22	0.74		
**Measure**									
SF12/36	6	0.46	0.37	0.07	0.85	0.08	1.13	0.967	0.436
NPI	2	0.32	0.14	−0.95	1.59	0.22	0.42		
Attitude	4	0.52	0.36	−0.06	1.09	0.30	1.06		
MMSE/MoCA	6	0.77	0.46	0.28	1.26	0.48	1.70		
**Frequency (times/week)**									
<3	4	1.04	0.48	0.28	1.79	0.65	1.70	6.030	0.012^*^
3~5	9	0.44	0.30	0.21	0.67	0.08	1.13		
>5	5	0.40	0.12	0.25	0.56	0.22	0.54		

* *p < 0.05*.

## Discussion

This study focused on the effects of aerobic exercise on the mental health of older adults (43). One-way ANOVA was used to examine four influencing factors across the study subjects, measures, intervention durations, and exercise frequency (44). The results show that only the ANOVA results were significantly different between different exercise frequencies. By contrast, there were no significant differences in the ANOVA results between the subjects, measurement indicators and intervention durations. This may not be in line with traditional studies. Therefore, we need to further analyze the possible reasons for this.

First, the quality of the included literature needs to be analyzed in terms of reliability. All the included studies were RCTs with the highest experimental grade, and all the studies were conducted in strict accordance with the established process for randomized controlled trials (45), except for four experimental designs with unclear random assignment methods and blinding points (11, 27, 33, 37). The included studies were reliable, with more than 70% to ensure a low risk of bias.

Second, was the coding of the impact factor classification scientific? The four impact factors selected for this study were reclassified and coded according to the needs of the study, and this classification was based on conventional experience (46). Therefore, the blind spots in the application of this method are currently unclear.

Finally, was the quality of the intervention effect size data extraction reliable? In addition to the categorical coding, the proposed intervention effect size is also an important factor influencing the results of the ANOVA in this study (47). Only one paper in this study provided effect size values directly (36), and the rest of the data were transformed using effect size calculation formula, which reduced the reliability of the data source. However, two people independently extracted and calculated the effect size separately, ensuring data integrity for the study. Despite all the three issues mentioned above, we followed strict scientific procedures to guarantee the quality of the included literature, coding classification and data extraction. However, the accuracy of the results provided by the original studies and the bias in the publication of the results could have affected the results of this study.

Comparison among the mean effect sizes of different exercise frequency groups (EFGs) showed that the lowest EFG obtained the largest effect size. The finding is similar to the results of a recent meta-analysis study of the cognitive function of older adults. That study suggested, in older adults, high-frequency exercise interventions did not affect cognitive function more than low-frequency ones (48). Similarly, another study of a 6-week exercise intervention showed no significant difference in effect size between the high-frequency and low-frequency groups (49). We reasoned that there might be methodological flaw in using only exercise frequency as an indicator of influencing factor. Yet another study revealed that exercise duration of more than 6 months was more effective than that of <6 months (50). In this study, the duration of the intervention was 6 months or more in 75% of the low-frequency group. Although the current evidence does not directly conclude that duration affects the effect of the intervention, regular and continuous exercise is undoubtedly beneficial for older adults. Thus, considering the benefits of low-frequency exercise with slightly higher or high-frequency exercise, older adults should primarily engage in low-frequency exercise.

In summary, there are several weaknesses in the present study. First, mixing different populations, outcome measures and exercise programs into the study may lead to high heterogeneity of fitting results. Second, one-way ANOVA only investigates the impact of a single factor on the observed variables, and cannot diagnose the interaction effects between factors (51). Third, selecting only effect size indicators ignores the value of sample size, which may produce uncontrollable errors (52). Therefore, future research should focus on seeking methodology breakthroughs while addressing the above issues.

## Conclusions

This retrospective study confirmed the positive effect of aerobic exercise on the mental health of older adults with a moderate overall intervention effect (ES Cohen's *d* = 0.56). The results of the one-way ANOVA revealed that adults over 60 years of age, regardless of whether they have an intellectual disability or not, or are undergoing physical rehabilitation or not (mild motor impairment), can improve their mental health through aerobic exercise. We recommend low-frequency exercise for older adults when the exercise benefits of various modes are compared.

## Author Contributions

LY, HF, WL, and JL: data collection. LY and JC: data analysis, conception, and design. LY, JL, and JC: research design, writing the manuscript, and revision. All authors contributed to the article and approved the submitted version.

## Funding

This study was funded by the National Education Science 13th Five-Year Plan Ministry of Education of the People's Republic of China Key Program (DLA190425).

## Conflict of Interest

The authors declare that the research was conducted in the absence of any commercial or financial relationships that could be construed as a potential conflict of interest.

## Publisher's Note

All claims expressed in this article are solely those of the authors and do not necessarily represent those of their affiliated organizations, or those of the publisher, the editors and the reviewers. Any product that may be evaluated in this article, or claim that may be made by its manufacturer, is not guaranteed or endorsed by the publisher.
