# Supplementary material for: Effect of Aerobic Exercise on Mental Health in Older Adults: A Meta-Analysis of Randomized Controlled Trials
Source: Front Psychiatry. 2021 Oct 6;12:748257. doi: 10.3389/fpsyt.2021.748257 (PMC8634786; doi:10.3389/fpsyt.2021.748257)
Supplement: Supplementary file 1 [file Table_1.DOCX]

**Supplementary Table 1. Risks Assessment of included studies**

| ID | Author, year | sequence generation | allocation concealment | participants blinding | assessor blinding | incomplete outcome data | selective outcome reporting |
| --- | --- | --- | --- | --- | --- | --- | --- |
| 1 | Anderson-Hanley2018 | H | L | U | U | L | L |
| 2 | Awick2015 | L | L | U | U | L | L |
| 3 | Bieler2017 | L | L | H | L | L | L |
| 4 | Cancela2016 | L | L | H | L | L | L |
| 5 | Cheung2017 | L | L | H | L | L | L |
| 6 | Eggenberger2015 | L | L | L | H | L | L |
| 7 | Hall2020 | L | H | H | L | L | L |
| 8 | Karssemeijer2019 | U | U | H | H | L | L |
| 9 | Langoni2019 | L | L | H | L | L | L |
| 10 | Middleton2018 | L | L | H | L | L | L |
| 11 | Parvin2020 | L | L | H | L | L | L |
| 12 | Suzuki2012 | U | U | U | U | L | L |
| 13 | Varela2018 | L | L | H | L | L | L |
| 14 | Wanderley2015 | L | L | H | L | L | L |
| 15 | Zanetidou2017 | L | L | H | L | L | L |

Note: Y=Low risk; N=High risk; U=Unclear.
